# Atrial Natriuretic Peptide and Adiponectin Interactions in Man

**DOI:** 10.1371/journal.pone.0043238

**Published:** 2012-08-16

**Authors:** Andreas L. Birkenfeld, Michael Boschmann, Stefan Engeli, Cedric Moro, Ayman M. Arafat, Friedrich C. Luft, Jens Jordan

**Affiliations:** 1 Department of Endocrinology, Diabetes and Nutrition, Center for Cardiovascular Research (CCR), Charité - University School of Medicine, Berlin, Germany; 2 Experimental and Clinical Research Center, a joint cooperation between the Charité Medical Faculty and the Max-Delbrück Center for Molecular Medicine, Berlin, Germany; 3 Inserm UMR1048, Institute of Metabolic and Cardiovascular Diseases, Université Paul Sabatier, Toulouse, France; 4 Institute of Clinical Pharmacology, Hannover Medical School, Hannover, Germany; Pennington Biomedical Research Center, United States of America

## Abstract

Reduced circulating natriuretic peptide concentrations are independently associated with insulin resistance and type 2 diabetes, while increased natriuretic peptide levels appear to be protective. Observations in vitro and in heart failure patients suggest that atrial natriuretic peptide (ANP) promotes adiponectin release, an adipokine with insulin sensitizing properties. We tested the hypothesis that ANP acutely raises adiponectin levels in 12 healthy men. We infused ANP intravenously over 135 minutes while collecting venous blood and adipose tissue microdialysates at baseline and at the end of ANP-infusion**.** We obtained blood samples at identical time-points without ANP infusion in 7 age and BMI matched men. With infusion, venous ANP concentrations increased ∼10 fold. Systemic and adipose tissue glycerol concentrations increased 70% and 80%, respectively (P<0.01). ANP infusion increased total adiponectin 14±5% and high molecular-weight (HMW)-adiponectin 13±5% (P<0.05). Adiponectin did not change in the control group (P<0.05 vs. infusion). ANP-induced changes in HMW adiponectin and adipose tissue lipolysis were directly correlated with each other, possibly suggesting a common mechanism. Our data show that ANP acutely increases systemic total and HMW-adiponectin concentrations in healthy subjects. Our study could have implications for the physiological regulation of adiponectin and for disease states associated with altered natriuretic peptide availability.

## Introduction

Reduced circulating natriuretic peptide concentrations are independently associated with insulin resistance and type 2 diabetes [1]–[3]. In contrast, augmented natriuretic peptide availability improves insulin sensitivity in mice [4]. Furthermore, genetic polymorphisms in the promoter region of the brain natriuretic peptide (BNP) gene are associated with increased BNP levels while protecting from type 2 diabetes [5]. How chronic changes in natriuretic peptides could affect glucose homeostasis in man is not understood. Atrial natriuretic peptide (ANP) and BNP effects on blood pressure and volume regulation have been extensively studied. However, natriuretic peptides also regulate adipose tissue metabolism. ANP and BNP induced natriuretic peptide receptor A activation potently stimulates adipose tissue lipolysis through cGMP and protein kinase G activation [6], [7]. The mechanism cannot explain protective natriuretic peptide influences on glucose metabolism. Instead, natriuretic peptide may promote adiponectin production, an adipokine with insulin sensitizing properties. ANP augmented adiponectin production and release from cultured human adipocytes [8]. In heart failure patients, therapeutic ANP infusions increased total and high molecular weight (HMW) adiponectin levels [9]. Studies in heart failure patients could be confounded by the underlying pathology. The heart failure-associated neurohumoral activation may be particularly important in this regard. Heart failure medications including beta-adrenoreceptor blockers and renin-angiotensin-aldosterone system inhibitors could also affect natriuretic peptide mediated responses. Therefore, we tested the hypothesis that ANP acutely increases adiponectin levels in healthy men.

## Methods

The local ethics committee approved the study and written-informed consent was obtained. We included 12 healthy men (30±2 years, 24.1±0.5 kg/m^2^) receiving no medications. After an overnight fast, we placed one catheter each into large antecubital veins of both arms. We used one catheter for infusion and the other one for blood sampling. We inserted a microdialysis probe (CMA/60 microdialysis catheters, Solna, Sweden, cut off 30 kDa) into abdominal subcutaneous adipose tissue to monitor changes in tissue lipolysis and blood flow (ethanol dilution). After at least 60 min resting phase, an incremental administration of human ANP (hANP) with a maximal rate of 25 ng/kg/min and a total infusion time of 135 min commenced as described previously [7] while blood pressure was closely monitored. ANP concentrations were determined using a radioimmunoassay. Total and HMW-adiponectin plasma concentrations were measured using multimeric ELISA. We monitored ANP-induced changes in adipocyte lipolysis through plasma and microdialysate glycerol measurements. To exclude a time effect, we also obtained venous blood samples in 7 healthy age and BMI-matched men (age 33±4 years, BMI 24±1 kg/m^2^) at identical time points without ANP infusion. Two tailed, one sample t-test and linear regression analysis were used to compare changes in adiponectin with ANP infusion and to establish associations between ANP, adiponectin and metabolic parameters, respectively. Changes between groups were compared by student’s t-test. Data are expressed as mean±SEM.

## Results

**Figure 1:**
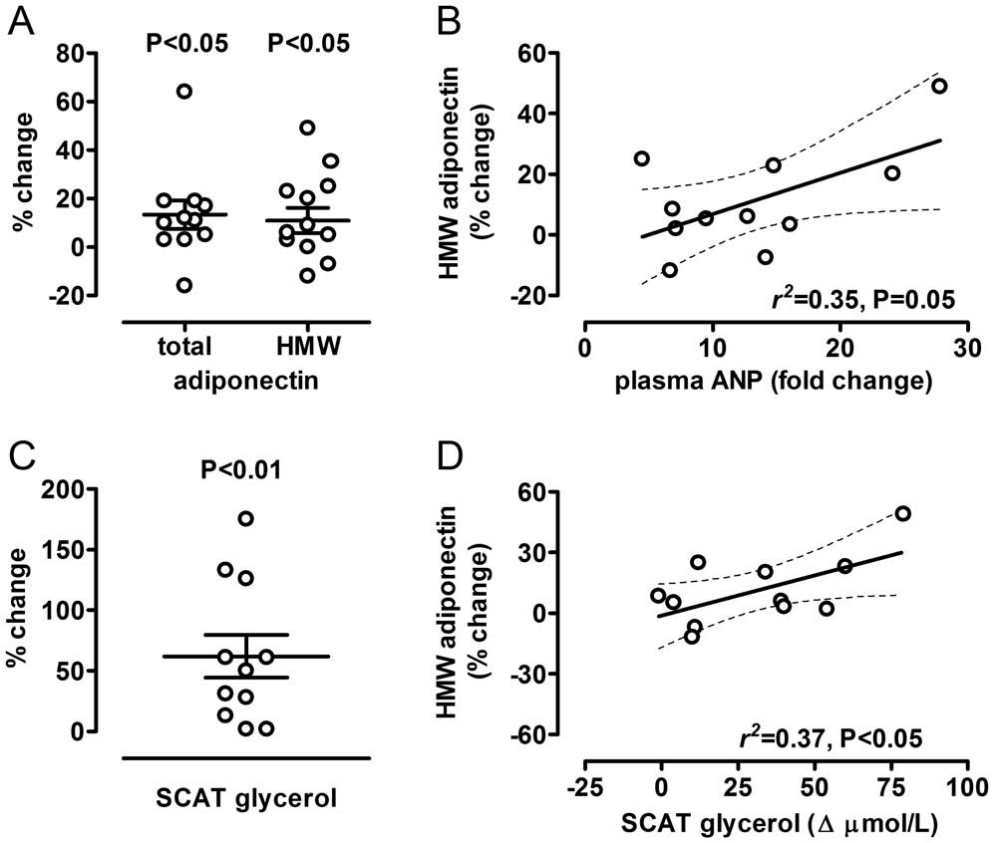
ANP induces total and HMW-adiponectin. A – Individual changes in venous total as well as HMW-adiponectin with ANP infusion. The bar indicates the mean value (P = compared to baseline, n = 12). B – Correlation between the change in plasma ANP and the change in HMW adiponectin with ANP infusion (n = 11). C – Individual changes in dialysate glycerol in subcutaneous adipose tissue (SCAT) with ANP infusion. The bar indicates the mean value (P = compared to baseline, n = 11). D - Correlation between Δ SCAT dialysate glycerol, a marker for lipolysis, and the change in HMW adiponectin with ANP infusion (n = 11).

Plasma ANP was 41±5 pg/mL at baseline and increased to 447±29 pg/mL at the end of the ANP infusion (P<0.01, data not shown). During ANP infusion, systolic blood pressure decreased from 116±3 mm Hg at baseline to 110±2 mm Hg at the end of ANP infusion (P<0.05). Diastolic blood pressure was 62±2 mm Hg at baseline and did not change significantly with ANP infusion (data not shown). Venous glycerol concentration increased from 48±5 µmol/L at baseline to 81±80 µmol/L with ANP infusion (P<0.01). Dialysate glycerol in adipose tissue increased from 51±6 µmol/L at baseline to 90±14 µmol/L with ANP infusion (p<0.01, figure 1) while the ethanol ratio did not change. Thus, ANP was sufficiently dosed to affect adipose tissue function. Total adiponectin was 5.6±0.5 pg/ml at baseline and increased by 14±5% (6.3±0.5 pg/ml, 95% CI from 2 to 25%, P<0.05) with ANP infusion (figure 1). HMW-adiponectin, the most potent isoform in terms of insulin sensitization, was 2.9±0.3 pg/ml at baseline and increased by 13±5% (3.49±0.4 pg/ml, 95% CI from 2 to 24%, P<0.05) with ANP (figure 1). The change in HMW-adiponectin was directly correlated with the change in plasma ANP with ANP infusion (r^2^ = 0.35, P = 0.05, figure 1). Changes in adipose tissue glycerol and HMW-adiponectin with ANP infusion also showed a positive correlation (r^2^ = 0.37, P<0.05, figure 1). In the control group, total adiponectin and HMW-adiponectin were reduced by 4±2% and 9±1%, respectively (ns), and the response was attenuated compared to the intervention group (P = 0.06 for total adipoenctin, P<0.05 for HMW-adiponectin).

To gain insight in mechanisms contributing to ANP-induced adiponectin regulation, we compared 4 subjects with the lowest adiponectin response (“non-responsive”) to the remaining 8 subjects (“responsive”) from the ANP treated group (figure 1 - panel A). We did not observe differences for ANP concentrations, glycerol plasma or dialysate concentrations as well as plasma free fatty acid-, norepinephrine or epinephrine concentrations (data not shown) between subgroups. However, waist circumference, reflecting intraabdominal fat mass, was 85±2 cm in the responsive and 91±2 in the non-responsive group (P = 0.056).

## Discussion

The main finding of our study is that ANP acutely increases systemic total and HMW-adiponectin concentrations in healthy subjects. Our study also suggests that ANP raises adiponectin in a concentration dependent fashion. The correlation between ANP induced adipose tissue lipolysis and adiponectin release might suggest a common transduction mechanism for both, lipase activation and adiponectin release involving adipocyte NPR-A receptors with subsequent cGMP generation. Our data is further supported by in vitro experiments in cultured human adipocytes showing that ANP dose-dependently enhanced the expression of adiponectin mRNA and its secretion from adipocytes [8]. However, we cannot completely rule out the possibility that ANP also altered renal or hepatic adiponectin clearance [10]. Our study could have implications for the regulation of adiponectin in conditions with altered natriuretic peptide availability. For instance, natriuretic peptides could mediate their effect on glucose metabolism through adiponectin, at least in a chronic setting, while acute ANP infusion did not result in an increase in insulin sensitivity [11]. Adiponectin is known to signal via the activation of AMP-activated protein kinase (AMPK). ANP has recently been shown to also activate AMPK in adipocytes [12]. We dare to suggest that natriuretic peptides might induce AMPK through adiponectin release.

Previous studies in heart failure patients showed that therapeutic ANP infusions for 3 days increased total and high molecular weight (HMW) adiponectin levels [9]. Patients with heart failure are characterized by specific traits: First, they have increased sympathetic nervous activity. Second, they have a whole range of co-morbidities, such as renal insufficiency and changes in body composition that interfere with natriuretic peptide levels, and finally, they receive different medications, such as beta-adrenergic receptor blockers, which also influence natriuretic peptide levels. The most important confounder in this regard is increased sympathetic nervous activity**.** Adipocyte stimulation with beta adrenergic receptor agonists potently reduced adiponectin expression and release [13]. The inhibitory response was almost completely reversed by non selective beta-adrenoreceptor blockade [13]. Natriuretic peptides reduce sympathetic nervous activity and the effect is more pronounced in heart failure patients compared to healthy controls [14]. Thus, in heart failure patients, adiponectin induction through natriuretic peptides could be in part explained by sympathetic inhibition, abolishing the inhibitory effect on adiponectin transcription and secretion. We have previously shown that the infusion of ANP in healthy lean subjects did not affect sympathetic nervous activity, and that ANP’s metabolic action in healthy subjects are not mediated through sympathetic mechanisms [15].

Plasma adiponectin as well as ANP concentrations are reduced in conditions associated with obesity [16]. Our preliminary subgroup analysis suggests that subjects with increased waist circumference, reflecting increased abdominal adiposity, might be less responsive to adiponectin induction through ANP. Indeed, increased adiposity is associated with reduced natriuretic peptide availability likely through increased clearance secondary to NPR-C scavenger receptor up-regulation in adipose tissue [17], [18]. Decreased natriuretic peptide availability at the adipose tissue level may promote adiponectin deficiency in this setting.

Paradoxically, increased adiponectin concentrations in heart failure and patients with myocardial infarction independently predict all cause and cardiovascular mortality [19]. Pathophysiological conditions including heart failure are associated with excessive ANP concentrations, which can increase up to levels as high as 500 pg/mL. Therefore, the levels induced in our study are of clinical relevance. We [20] and others [21] have shown that in heart failure patients, adipose tissue does not seem to desensitize towards metabolic natriuretic peptide actions. Thus, natriuretic peptides could chronically raise adiponectin concentrations, particularly in severely affected heart failure patients. The mechanisms could explain the counterintuitive direct relationship between adiponectin levels and mortality in cardiac patients. We conclude that natriuretic peptides modulate systemic adiponectin concentrations in young healthy individuals, thus, providing a link between cardiac function, volume status, and lipid and glucose metabolism.
